# Aging and Cancer—Inextricably Linked Across the Lifespan

**DOI:** 10.1111/acel.14483

**Published:** 2025-01-21

**Authors:** James DeGregori, Katherine J. Seidl, Monty Montano

**Affiliations:** ^1^ Department of Biochemistry and Molecular Genetics University of Colorado School of Medicine Aurora Colorado USA; ^2^ Global Head Oncology Drug Discovery Unit Takeda Pharmaceuticals Cambridge Massachusetts USA; ^3^ Department of Medicine Harvard Medical School Boston Massachusetts USA

## Abstract

Aging (as old man wind) alters the trajectory of cancer (dangerous seas) through changes in the immune system and metabolism (among many others), leading to altered cancer epidemiology, pathogenesis, and therapeutic responses, as represented by the research areas (boats)—artwork by Michael DeGregori.

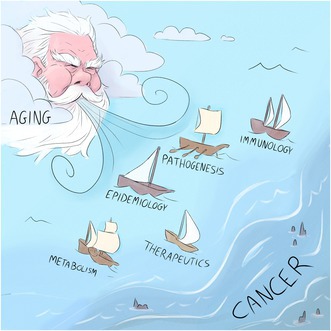

## 1 Introduction

The convergence of aging and cancer opens significant avenues for biomedical research. Aging profoundly impacts cancer risk and treatment outcomes, highlighting the necessity of considering biological age in cancer studies. Factors, such as an aging immune system, lifetime environmental exposures, vaccination histories, biological and psychosocial stress, and physiological reserves, are pivotal in cancer development and progression throughout an individual's lifespan.

An interdisciplinary approach is crucial for integrating insights from aging and cancer biology. This requires collaboration across fields, such as geroscience, physiology, molecular epidemiology, biochemistry, and drug response, to advance understanding and develop new treatments.

Progress in age‐related cancer research, prevention, and therapy necessitates partnerships among government, philanthropic organizations, biotech and pharmaceutical companies, and academic publishers. These collaborations can drive innovation and accelerate advancements in the field.

In response, four journals—*Aging Cell*, *Advanced Biology*, *Aging & Cancer*, and *Advanced Science—*will host a virtual special collection of new and previously published studies focused on cancer and aging. This initiative aims to encourage research exploring the relationship between aging and cancer, building awareness of their interconnected influence on cancer development, growth, and metastases.

The objective is to inspire research that leads to cancer treatment strategies tailored to different age groups, reducing the cancer burden across diverse populations and health conditions. By focusing on the intricate connection between aging and cancer, the biomedical community can contribute to more effective cancer prevention, detection, and treatment, thereby reducing the cancer burden across the lifespan.

This editorial advocates for studies that adopt a more personalized approach to cancer treatment, considering the unique interplay of age‐related factors, comorbid conditions, and individual patient histories to optimize cancer prevention and therapeutic outcomes. We will provide a brief overview of how aging alters cancer risk and pathogenesis and provide our perspective on the exciting directions possible for research into the interface of aging and cancer.

## 2 Navigating the Aging Influence

Adults aged 65 years and above account for over half of all cancer diagnoses in the United States (An et al. 2023). Clearly, there is a pressing need to explore how aging affects cancer. Despite advances in cancer treatment, outcomes vary widely by cancer type, age, race, ethnicity, and socioeconomic status, highlighting the complexity of cancer and the factors that influence disease progression.

Age plays a crucial role in the onset of clinical cancer, influenced by genetics, lifestyle, and environmental exposures. The changing patterns of cancer incidence evident at different life stages also mirror shifts in overall health, resilience, and immune function, which are critical to understanding cancer progression.

Another example of age‐related changes with relevance to cancer progression and treatment is the tissue microenvironment, which includes heterogeneous cell types based on the details of organ function and health status, and an extracellular matrix (ECM) that remodels with age. Notably, increased dysregulation in ECM associated with age influences tumor progression and clearance by enhanced susceptibility to metastatic cancer cell colonization (Deasy and Erez 2022). Indeed, characteristics of the tumor ECM are closely correlated with disease progression, resistance to therapy, and poor prognosis (Marino and Weeraratna 2020).

Although the fields of aging and cancer biology have advanced, the integration of these fields is in its infancy, offering opportunities for groundbreaking insights. The interplay between aging and cancer extends to how changing immunity and tissues sculpt cancer evolution, and how the cancer sculpts the tumor microenvironment, influencing cancer evolutionary trajectories through cell intrinsic (e.g., mutations) to extrinsic (microenvironmental) changes, impacting everything from cancer incidence to treatment outcomes.

Recent research has shed light on organ cross talk, the impact of immune aging on tumor surveillance, and the significance of organ senescence and clonal hematopoiesis (Marongiu and DeGregori 2022; Fane and Weeraratna 2020). This knowledge paves the way for developing more effective, age‐specific cancer treatments, promising a future where cancer care is tailored to the complexities of aging and health trajectories.

## 3 Aging and Cancer Risk

**FIGURE 1:**
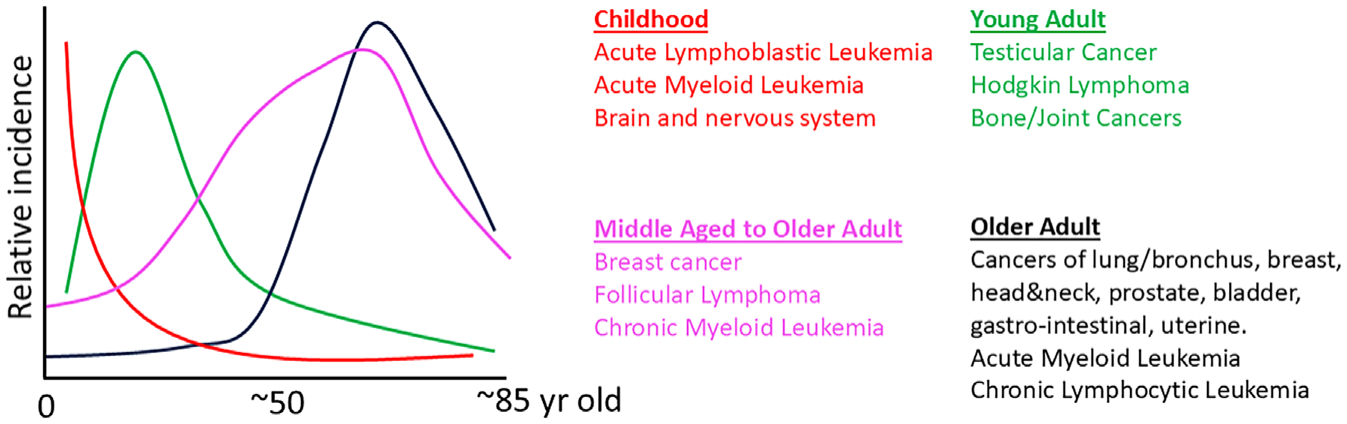
The incidence of different cancers varies substantially at different ages. The curves drawn are derived from SEER data for new cases (see Figure S1 and https://seer.cancer.gov/). Curves for each of the four subgroups of cancer are drawn for representative cancer—acute lymphoblastic leukemia for Childhood, testicular cancer for Young Adult, breast cancer for Middle Aged to Older Adult, and lung/bronchus cancers for Older Adult. Thus, these curves should not be considered precise for the cancers of each age period. Also, note that graphs are based on the percentage of new cases (see Figure S1), and thus reductions at the oldest ages in part reflect reduced representation of these age groups in the populations.

**FIGURE 2:**
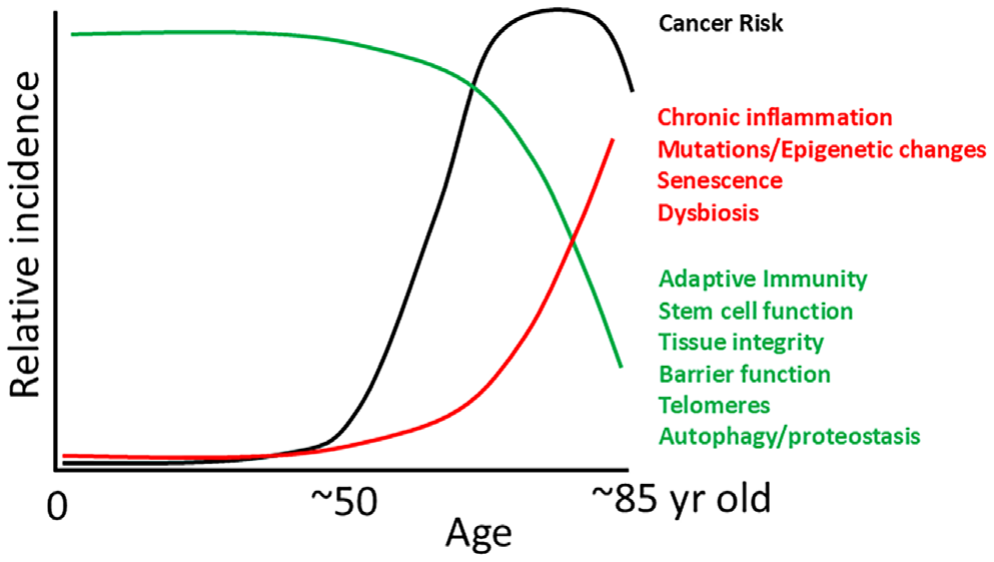
Physiological changes that accompany and contribute to the increase in cancer risk with age. The increasing risk of cancer is accompanied by the decline in key features of tumor suppression (e.g., adaptive immune function and tissue maintenance programs) and increases in tumor promoting factors (e.g., chronic inflammation and dysbiosis).

The link between aging and cancer risk is complex, significantly influenced by both age and a mix of genetic and environmental factors. We observe a gradual shift from early‐life cancers, such as childhood leukemias, neuroblastoma, glioblastoma, and retinoblastoma, to early adult cancers like lymphomas and testicular cancer, and then older adult‐onset diseases such as breast, lung, gastric, and prostate cancer, providing evidence that cancer risk in different tissues evolves over a person's lifespan (Figure 1). While some cancers, such as leukemias and glioblastomas, occur at both the youngest and oldest ages, the biology and genetics of these cancers are quite distinct at these extremes of our lifespans. Indeed, the burden and types of mutations change substantially for cancers diagnosed at different ages (Chatsirisupachai, Lagger, and de Magalhães 2022). Changes in cancer susceptibility, accompanied by substantial differences in therapeutic outcomes, parallel other aging‐associated changes in tissue development and maintenance, including our immune systems (Figure 2).

With the median age of cancer diagnosis at 66 years (cancer.gov), advancing age is one of the most important risk factors for cancer. Aging introduces a dual challenge: an increase in genetic mutations and decreases in our evolved defenses against cancer evolution in postreproductive years (Marongiu and DeGregori 2022), with vulnerability compounded by accumulated health issues. This sensitivity is heightened by both intrinsic factors, like epigenetic changes and immune dysregulation, and extrinsic ones, such as lifestyle and environmental exposures (Podolskiy and Gladyshev 2016). Emerging tools like biological aging clocks, using biomarkers from DNA methylation to proteomics, offer new ways to understand how age‐related changes contribute to cancer. Metabolic program dysregulation, epigenetic drift, and inflammaging in the microenvironment may act as cancer drivers and need more mechanistic clarity (Verovskaya, Dellorusso, and Passegue 2019). The precise mechanisms—both intrinsic and extrinsic—behind these links to cancer initiation and progression are areas of active investigation.

Deciphering the complex dynamics between aging, genetic predispositions, and environmental influences on cancer risk presents a significant opportunity for innovation in cancer research. By focusing on these intersections, research can pave the way for advancements in cancer prevention and treatment that are finely tuned to diverse life experiences.

## 4 The Response to Therapy: Age Matters

As patients age, several factors evolve that can profoundly influence cancer progression and responses to therapies. These factors include immune system changes, environmental exposures over a lifetime (exposome), frailty, the cumulative impact of stress (i.e., allostatic burden), comorbidities, and the varying degrees of physical and psychosocial resilience that come with aging and lived experiences. Additionally, a patient's treatment history and the secondary effects of those treatments on organ function play a crucial role in determining the efficacy of future therapies and could be pivotal in tailoring treatment options. Furthermore, hematological indications such as leukemias and lymphomas are more readily accessible for such analyses relative to solid tumor indications (Morita et al. 2020) and represent powerful opportunities to understand the evolutionary process of therapeutic resistance. Notably, the age‐dependent expansions of clones (often bearing cancer‐associated mutations) in our tissues (Marongiu and DeGregori 2022), which are associated with both malignant and nonmalignant disease risk as shown for the hematopoietic system (Jaiswal and Ebert 2019; Florez et al. 2022), still represent a relatively unexplored frontier, particularly regarding the impact of these clonal expansions on patient responses to therapies and overall well‐being.

Because the immune system undergoes change throughout the life course (e.g., age‐related decline in naïve CD8^+^ T cells and expansion/exhaustion of memory phenotypes; increasing presence of GMZK^+^ CD8^+^ T cells; and biased expansion of myeloid‐to‐lymphoid cells; Connolly et al. 2024; Luo et al. 2022; Mogilenko et al. 2021) the function of immunotherapies such as checkpoint inhibitors and CAR‐T cells, as well as immune‐related adverse events, may vary accordingly. In infants and children, while CAR T cells have demonstrated success for B‐cell acute lymphocytic leukemias, immune checkpoint inhibitor therapies have been less effective, likely due to the low mutation burden of pediatric cancers (Long et al. 2022). Older and geriatric adults who are experiencing immune systemic and cellular senescence changes associated with aging still exhibit responses to checkpoint inhibitors (Hamilton and Henry 2020) (Zhang et al. 2022), and CAR‐T cell therapy can still be effective against B‐cell lymphomas (Chihara et al. 2023), albeit with reduced responses in those over 75. For older persons, factors such as prior antigen exposure and overall health status should clearly play roles but currently are understudied.

Approaches, such as systems serology (Chung and Alter 2017), that define prior immune exposures and responses, along with cell phenotyping to assess functional immune responses, or ex vivo immune challenge, could be leveraged to guide the choice and predicted outcomes of immunotherapies. Understanding age‐related influences on immunotherapy efficacy will be crucial for ensuring effective treatment across diverse populations. Additional immunotherapeutic opportunities include identifying approaches to limit the accumulation of senescent cells and exhausted cells; limiting genotoxic stress and radiation treatment induced DNA damage and senescence; as mentioned earlier, overcoming tissue contextual changes in the ECM within the tumor microenvironment that often limit access to tumors; adapting immunotherapies given the age‐related increases in PD1 expression on T cells and age‐related changes in metabolites (e.g., methylmalonic acid; Gomes et al. 2020); and anticipating treatment‐related complications and comorbidities, such as frailty. Further to this point, aging is often accompanied by the accumulation of both subclinical and clinical comorbid conditions, which can alter treatment responses and influence disease progression. Different comorbidities (e.g., metabolic disease, cardiovascular disease, inflammatory syndromes) can modulate the pathophysiology of cancer through shared risk factors and biological pathways such as inflammation and immune function (Renzi et al. 2019). Therefore, acknowledging and addressing these comorbidities is essential in crafting effective, patient‐specific treatment plans.

Lastly, ensuring equitable access to cancer care throughout an individual's life is crucial for extending health span and improving outcomes. The disparities in access to care, influenced by various social and economic factors, must be addressed to provide all patients with the opportunity for optimal treatment and care, regardless of age or background.

## 5 Fostering Research at the Cancer–Aging Interface

As we delve into the complex interplay between aging and cancer, a pivotal question arises: should we reshape our funding strategies for cancer diagnostics and treatment to better address the interplay between biological aging and age‐related comorbid conditions? Should we encourage a further shift toward interdisciplinary programs that bridge the gaps between institutes like the National Institute on Aging (NIA) and the National Cancer Institute (NCI), fostering collaborative efforts that can accelerate breakthroughs in understanding and treatment? As our appreciation of the interconnectedness of different tissues and diseases grows, a more holistic view of the pathogenesis of cancer and other diseases should help break down the silos that have historically isolated different disease‐focused fields.

Moreover, the potential benefits of establishing a seamless pipeline between the National Institutes of Health (NIH) and industry stakeholders for drug development are becoming increasingly clear. Such collaborations could streamline the process of bringing innovative treatments from the laboratory to the patient, particularly for age‐specific cancer therapies.

With cancer survival rates improving, we must also turn our attention to optimizing the quality of life for survivors. As cancer and aging begin to be viewed more as chronic conditions, requiring patients to transition from one medication to another, it is evident that our funding models for medication development and approval must evolve. This includes strategizing the development of therapies tailored to various age groups, ensuring that treatment is effective and appropriate for each stage of life in a person's health trajectory. We must also address issues with the delivery of care that are impacted by age (intersecting with other disparities), such as medication adherence, transportation to appointments, and the need for care givers. Importantly, we need to correct the striking underrepresentation of older persons in clinical trials (Dotan 2017).

Utilizing the vast data available from aging cohorts and cancer research, encompassing both model organisms and human studies is crucial for uncovering potential connections and dependencies between cancer and aging biology. We also see value in inclusive clinical trial design that better represents the diversity of the population, particularly older individuals, and accounts for sex, gender, and genetic background variability. Although there is increasing awareness of the need to ensure clinical trials include a diverse patient population, currently, these demographic factors can be underrepresented, undermining the universality and applicability of research findings.

Supporting research at the intersection of aging and cancer calls for a holistic approach that includes adjusting funding strategies, fostering interdisciplinary collaborations, rethinking clinical trial inclusivity, and innovating biological models. This multifaceted strategy is essential for advancing our understanding and treatment of cancer in the context of aging, paving the way for more effective and personalized care across the lifespan.

## 6 Challenges and Opportunities Ahead

**FIGURE 3:**
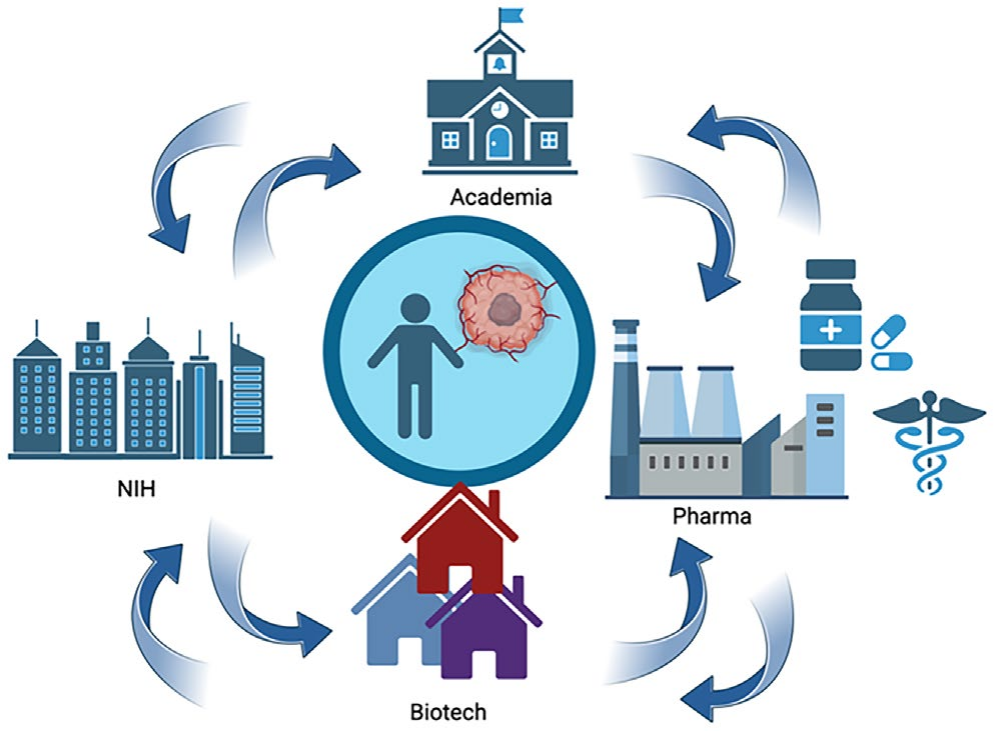
Collaborative scenarios fostering partnerships in the ecosystem dedicated to cancer and aging. Interactions and partnerships between NIH Centers, Biotech, Academic institutions, and Pharmaceutical Industries can enhance research and therapeutic development for aging patients with cancer. Created in Biorender.

Research at the interface of cancer and aging presents a landscape filled with both significant challenges and promising opportunities. The traditional “stay in your lane” mentality, characterized by silos within the scientific community, severely limits the potential for groundbreaking innovation and discovery in cancer. There is a growing consensus on the need for more integrative approaches that foster partnerships across departments, NIH centers, pharmaceutical companies, and industries (Figure 3).

One of the key challenges in this area is the increased cost and time associated with studying aging, particularly with aged animal models. Innovative and clinically relevant biological models are needed. Traditional studies often rely on young mouse models, which may not accurately represent the disease in older mice let alone humans. Exploring alternative nonanimal models, such as human organoid systems that mimic the tumor microenvironment or tissue‐on‐a‐chip approaches, could offer more cost‐effective and time‐efficient research avenues. Additionally, living systems such as short‐lived killifish present an innovative method to study aging in a more controlled and scalable manner, which alongside comparative and evolutionary studies (e.g., cancer rarity in naked mole rats), could offer deeper insights into cancer resistance mechanisms and aging (Seluanov et al. 2018).

As cancer survivors live longer, there is also a clear need to adapt therapeutic strategies across the life course, considering the emergence of new morbidities over the life course. The pharmaceutical and biotech industries play a crucial role in the translation of discoveries into effective therapies.

The cancer and aging interdisciplinary field is gaining traction, underscored by these topics being presented at recent national and international meetings, along with special issues in other leading journals. Numerous institutions and universities have launched initiatives focused on the intersection of aging and cancer, many within the last decade. Enhancing communication and linkage among these initiatives could propel research in cancer and aging forward. The time may have come for the NIH to leverage this growth by considering a “Common Fund” approach to foster these connections.

With scientific endeavors on the cusp of various AI (artificial intelligence)/ML (machine learning) approaches, the area of cancer and aging is ripe for impact in this discipline. Recent analyses indicate that dominant aggressive cancer clones are present in the body at a very young age (Nishimura et al. 2023). To be able to understand how such clones evolve in each patient given the biology of gene–to‐gene, gene–to‐drug, and gene–to‐microenvironment interactions over the lifespan of a given patient requires a broad range of in vitro and in vivo model systems to generate data that both informs and predicts. Somatic mutations present in adulthood can be used to reconstruct the phylogeny of cancer cell development and dominance before, during, and after treatment and across the lifespan. Using different molecular methodologies such as molecular clocks of aging and cell lineage tracing (Gabbutt et al. 2022; Massaar and Sanders 2023) can enable the analysis of clonal expansion as markers of aging (Massaar and Sanders 2023). Better methods are needed to understand these expansions and the interplay with age‐related increase of inflammation and possible age‐related deterioration of the tissue microenvironment.

The collective movement in this field motivates this call to action, encouraging submissions to our upcoming virtual issue on cancer and aging. By embracing interdisciplinary collaboration, leveraging innovative models, and addressing the unique challenges of this research area, the scientific community can uncover new pathways to understanding and treating cancer in the context of aging, leading to improved outcomes and quality of life across the lifespan.

## Author Contributions

J.D., K.J.S., and M.M. jointly wrote the manuscript, from conception to editing.

## Conflicts of Interest

K.J.S. is a full‐time employee of Takeda Development Center Americas Inc. (TDCA), Lexington, MA, USA. M.M. is an Editor‐in‐Chief of *Aging Cell*, and J.D. is an Editor‐in‐Chief of *Aging and Cancer*.

## Supporting information


**Figure S1.** The percentage of new cases for each of the indicated cancers was graphed using the SEER web resource at https://seer.cancer.gov/.

## Data Availability

I believe Editorials should be exempt. We plot data from SEER, which is publicly available.
