# Supplementary figures and images for: Aging and Cancer—Inextricably Linked Across the Lifespan
Source: Aging Cell. 2025 Jan 21;24(4):e14483. doi: 10.1111/acel.14483 (PMC11984663; doi:10.1111/acel.14483)

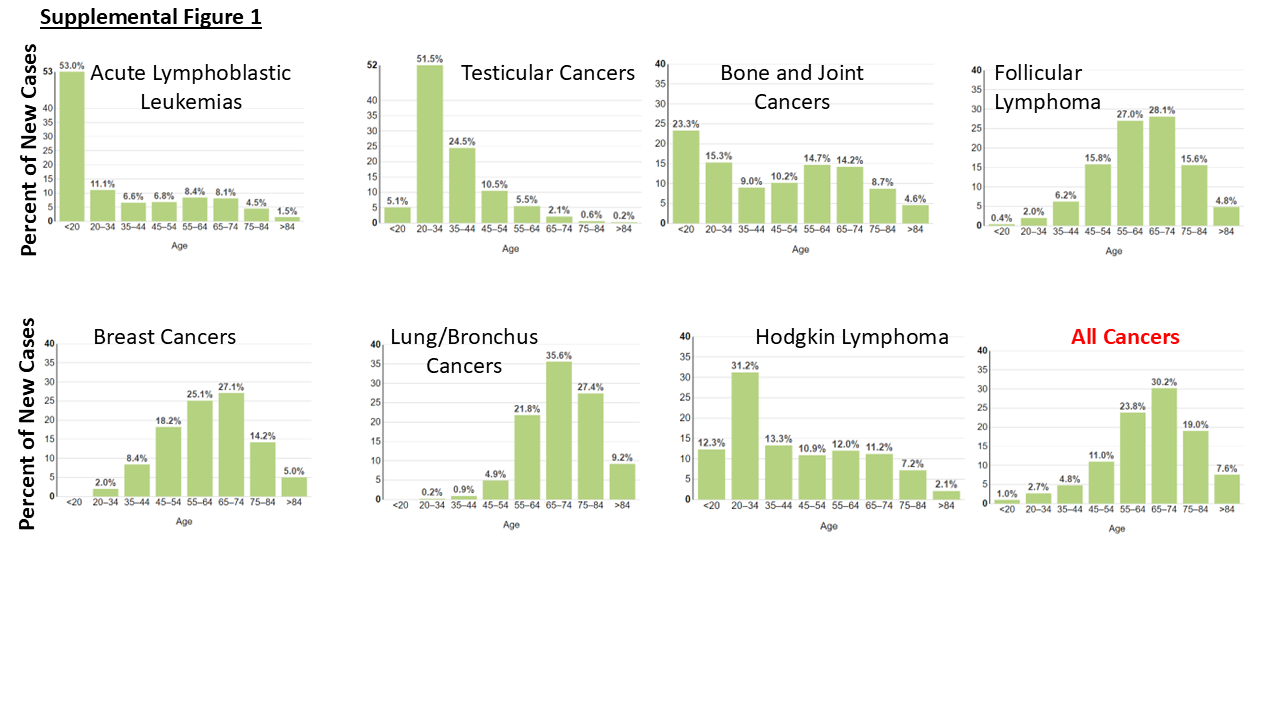

Supplement: Supplementary file 1 — Figure S1. The percentage of new cases for each of the indicated cancers was graphed using the SEER web resource at https://seer.cancer.gov/. [file ACEL-24-e14483-s001.png]
